# TME17/473: Web-Based Visualization and Processing of Anthropometric Data

**DOI:** 10.2196/jmir.1.suppl1.e124

**Published:** 1999-09-19

**Authors:** P Lesný, J Vejvalka, H Krásnièanová

**Affiliations:** ^1^2nd Medical FacultyCharles UniversityPrahaCzech Republic

**Keywords:** Telemedicine, Internet, Growth Disorders

## Abstract

**Introduction:**

Body height and body weight are the most common anthropometric values that are observed in children by their parents. Basic anthropometric measurements are also a common and natural part of routine medical check-ups in infants, children and adolescents. Although commonly taken, this data is not always correctly interpreted: cases when anthropometric data might have served for an earlier diagnosis of e.g. an endocrine disorder still occur. To facilitate utilization of this data, a software module was developed that visualizes the data and also provides a basic interpretation of the curves obtained. This software module consists of the knowledge base which was written at the auxological department of 2nd Medical Faculty and its interpreter. This platform independent module is offered to software developers for integration into their GP systems.

**Methods:**

To demonstrate the functions of the module offered to software developers and also to allow patient (and parent) growth self-assessment, the module that visualizes and interprets children's anthropometric data is ported into a WWW interface. Each pro-band is assigned a unique (and anonymous) ID, so that new data can be added in the course of time. Various anthropometric parameters can be followed - so that the system can serve not only parents to assess their children's growth, but also can help the practitioner to visualize and evaluate more intrinsic relations in children's development.

**Results:**

The service is just being launched at the web site of the 2nd Medical Faculty of Charles University - so the most important result of the project is the function of the system itself, that allows for further refinements of the project. The WWW interface also allows extensive testing of the module and demonstration of the module's capabilities to the software developers, GPs and other potential users.

**Discussion:**

During the future project development will be necessary to refine various components that surround the WWW portation of the core anthropometric module as the user interface, security issues, data storage etc.. We believe that the project will be found useful by parents and GPs, and we hope that it will also bring us interesting data for further analyses. We believe that this specialized software module developed by experts and integrated into the GP software systems will be a big step towards improvement of the computer assisted growth diagnosis.

